# Conceptualising and measuring social media engagement: A systematic literature review

**DOI:** 10.1007/s43039-021-00035-8

**Published:** 2021-08-11

**Authors:** Mariapina Trunfio, Simona Rossi

**Affiliations:** Department of Management and Quantitative Studies, University of Naples “Parthenope”, Naples, Italy

**Keywords:** Customer engagement, Social media engagement, Social media platforms, Qualitative metrics, Quantitative metrics, Social media metrics, COBRA model

## Abstract

The spread of social media platforms enhanced academic and professional debate on social media engagement that attempted to better understand its theoretical foundations and measurements. This paper aims to systematically contribute to this academic debate by analysing, discussing, and synthesising social media engagement literature in the perspective of social media metrics. Adopting a systematic literature review, the research provides an overarching picture of what has already been investigated and the existing gaps that need further research. The paper confirms the polysemic and multidimensional nature of social media engagement. It identifies the behavioural dimension as the most used proxy for users' level of engagement suggesting the COBRA model as a conceptual tool to classify and interpret the construct. Four categories of metrics emerged: quantitative metrics, normalised indexes, set of indexes, qualitative metrics. It also offers insights and guidance to practitioners on modelling and managing social media engagement.

## Introduction

Over the last decade, customer engagement has received increasing attention in academic and professional debate (Hollebeek, 2019; Kumar et al., 2019; Marketing Science Institute, 2020; Peltier et al., 2020; Rather et al., 2019; Rossmann et al., 2016). It can be considered a “consumer’s positively brand-related cognitive, emotional and behavioural activity during, or related to, focal consumer/brand interactions” (Hollebeek, 2014, p.149). Engaged customers display greater brand loyalty and satisfaction (Bowden, 2009; Jaakkola & Alexander, 2014) and are more likely to contribute to new product development (Haumann et al., 2015), service innovation (Kumar et al., 2010), and viral marketing activity spread by word of mouth (Wu et al., 2018). Customer engagement can also be linked with important brand performance indicators, including sales growth, feedback, and referrals (Van Doorn et al., 2010).

Acknowledging the potential of ICTs, scholars and practitioners are experimenting with new ways to capitalise on customer engagement and adapt to the new challenges of digital platforms (Barger et al., 2016; Peltier et al., 2020). Social media platforms reshaped the dyadic interaction between customers and organisations, creating spaces for digital sharing and engagement. By enabling users to comment, review, create, and share content across online networks, social media provide direct access to brands and allow co-creation processes. As such, the pervasive character of social media with its potential for engaging with customers and building relationships generated much interest in the concept of social media engagement (Barger et al., 2016; Hallock et al., 2019; Oviedo-García et al., 2014; Peltier et al., 2020; Schivinski et al., 2016). Engaging with customers in real-time and managing many incoming customers’ big data interested academic investigation and opened opportunities for marketers to enhance social media marketing success (Liu et al., 2019).

Understanding, monitoring, and measuring social media engagement are key aspects that interest scholars and practitioners who proposed diverse conceptualisations, several indicators and KPIs. With the spread of social media analytics, social networking platforms, digital service providers, marketers, and freelancers developed their metrics to measure engagement with brand-related social media contents and advertising campaigns. At the same time, scholars have pointed out various metrics and procedures that contribute to evaluating social media engagement in different fields (Mariani et al., 2018; Muñoz-Expósito et al., 2017; Trunfio & Della Lucia, 2019). Nevertheless, many of these studies offer a partial perspective of analysis that does not allow the phenomenon to be represented in diverse aspects (Oviedo-García et al., 2014). As a result, social media engagement remains an enigma wrapped in a riddle for many executives (McKinsey, 2012). How communities across an ever-growing variety of platforms, new forms of customer-brand interactions, different dimensions and cultural differences impact social media engagement measurement represents one of the main challenges (Peltier et al., 2020).

Although social media engagement represented a key topic in marketing research (Barger et al., 2016; Peltier et al., 2020), an overarching perspective of the existing knowledge can drive the investigation of the state of the field, including the study of the research streams, and the analysis of the measurement tools. This paper aims to systematically contribute to the academic debate by analysing, discussing, and synthesising social media engagement literature from the social media metrics perspective. A systematic literature review approach provides an overarching picture of what has already been investigated and the existing gaps that need further research. It contributes towards a systematic advancement of knowledge in the field and offers insights and guidance to practitioners on modelling and managing social media engagement (Tranfield et al., 2003).

The remainder of the paper is structured as follows. Section 2 presents the theoretical background of the study on customer engagement and social media engagement. Section 3 describes the methodology used for conducting the systematic literature review (Pickering & Byrne, 2014; Tranfield et al., 2003). Section 4 presents the bibliometric analysis results, including the year in which research began, the journals that publish most research, and the most relevant authors with publications on the topic. Then, Sect. 5 classifies these studies in terms of four macro-themes, conceptualisations, platforms, measurement, and behaviours and describes the key results available in the literature. Section 6 provides a critical discussion of the findings from the literature review and highlights its key contributions. Lastly, Sect. 7 concludes the study by highlighting its limitations and proposing directions for future research.

## Theoretical background

### Customer engagement

Although customer engagement research has increased theoretical and managerial relevance (Brodie et al., 2011; Hollebeek et al., 2016, 2019; Kumar et al., 2019; Vivek et al., 2012), to date, there is still no consensus on its definition due to its multidimensional, multidisciplinary and polysemic nature.

Several customer engagement conceptualisations have been proposed in the literature, drawing on various theoretical backgrounds, particularly service-dominant logic, and relationship marketing. From a psychological perspective, one of the first definitions of customer engagement is the one of Bowden (2009) that conceptualises it as a psychological process that drives customer loyalty. Similarly, Brodie et al. (2011) define customer engagement as a psychological state that occurs by interactive, co-creative customer experiences with a focal object. Later, focusing on the behavioural aspects, it has been described as the intensity of an individual’s participation in an organisation’s offerings or organisational activities (Vivek et al., 2012). More recently, from a value-based perspective, customer engagement has been defined as the mechanics that customers use to add value to the firm (Kumar et al., 2019).

Although the perspectives may vary, common elements can be identified in various conceptualisations. Literature generally understands customer engagement as a highly experiential, subjective, and context-dependent construct (Brodie et al., 2011) based on customer-brand interactions (Hollebeek, 2018). Moreover, scholars agree on its multidimensional nature (Brodie et al., 2013; Hollebeek et al., 2016; So et al., 2016; Vivek et al., 2012) encompassing cognitive (customer focus and interest in a brand), emotional (feelings of inspiration or pride caused by a brand), and behavioural (customer effort and energy necessary for interaction with a brand) dimensions. Also, researchers have proposed that customer engagement affects different marketing constructs (Brodie et al., 2011; Van Doorn et al., 2010). For example, in Bowden’s research (2009), there is evidence to support that customer engagement is a predictor of loyalty. Brodie et al. (2011) explore its effects on customer satisfaction, empowerment, trust, and affective commitment towards the members of a community. Van Doorn et al. (2010) propose customer-based drivers, including attitudinal factors such as satisfaction, brand commitment and trust, as well as customer goals, resources, and value perceptions.

### Social media engagement: The academic perspective

Social media engagement has also been investigated as brand-user interaction on social media platforms (Barger et al., 2016; De Vries & Carlson, 2014; Hallock et al., 2019; Oviedo-García et al., 2014; Peltier et al., 2020; Schivinski et al., 2016). However, while conceptual discussions appear to dominate the existing customer engagement literature, research results fragmented when moving to the online context. Scholars agree that social media engagement is a context-specific occurrence of customer engagement (Brodie et al., 2013) that reflects customers’ individual positive dispositions towards the community or a focal brand (Dessart, 2017). Social media engagement can emerge with respect to different objects: the community, representing other customers in the network, and the brand (Dessart, 2017). Furthermore, antecedents and consequences of social media engagement have been identified to understand why customers interact on social media and the possible outcomes (Barger et al., 2016), such as loyalty, satisfaction, trust, and commitment (Van Doorn et al., 2010).

In continuity with literature on customer engagement, also social media engagement can be traced back to affective, cognitive, and behavioural dimensions (Van Doorn et al., 2010). Most of the literature focuses on the behavioural dimension as it can be expressed through actions such as liking, commenting, sharing, and viewing contents from a brand (Barger et al., 2016; Muntinga et al., 2011; Oh et al., 2017; Oviedo-García et al., 2014; Peltier et al., 2020; Rietveld et al., 2020; Schivinski et al., 2016). It is worth pointing out that not all these actions determine the same level of engagement. Schivinski et al. (2016) in the COBRA (Consumer Online Brand Related Activities) Model differentiate between three levels of social media engagement: consumption, contribution, and creation. Consumption constitutes the minimum level of engagement and is the most common brand-related activity among customers (e.g., viewing brand-related audio, video, or pictures). Contribution denotes the response in peer-to-peer interactions related to brands (e.g., liking, sharing, commenting on brand-related contents). Creation is the most substantial level of the online brand-related activities that occur when customers spontaneously participate in customising the brand experiences (e.g., publishing brand-related content, uploading brand-related video, pictures, audio or writing brand-related articles). Starting from these social media actions, scholars attempted to measure social media engagement in several ways developing scales, indexes, and metrics (Harrigan et al., 2017; Oviedo-García et al., 2014; Schivinski et al., 2016; Trunfio & Della Lucia, 2019). Nevertheless, many of these studies offer a partial perspective of analysis that does not allow the phenomenon to be represented in its diverse aspects (Oviedo-García et al., 2014). Researchers have also examined emotional and cognitive dimensions (Dessart, 2017) as essential components of social media engagement that lead to positive brand outcomes (Loureiro et al., 2017).

### Social media engagement: The practitioners’ perspective

In business practice, the concept of customer engagement appeared for the first time in 2006 when the Advertising Research Foundation (ARF), in conjunction with the American Association of Advertising Agencies and the Association of National Advertisers, defined it as a turning on a prospect to a brand idea enhanced by the surrounding context (ARF, 2006)*.* Later, several consulting firms tried to give their definition emphasising different aspects and perspectives. For example, in 2008, Forrester Consulting, an American market research company, defined customer engagement as a way to create *‘deep connections with customers that drive purchase decisions, interaction, and participation over time’* (Forrester Consulting, 2008, p.4). Gallup Consulting identified four levels of customer engagement and defined it as an emotional connection between customers and companies (Gallup Consulting, 2009). Similarly, the famous American software provider Hubspot (2014) identified social media engagement as ‘*the ongoing interactions between company and customer, offered by the company, chosen by the customer’* (Hubspot, 2014, p.1).

With the increasing spread of social networks and their exploitation as an important marketing tool, practitioners recognised a clear linkage between customer engagement and the metrics to assess digital strategy success. Over time, social networking platforms such as Facebook, LinkedIn, and YouTube, developed their metrics to measure engagement with brand-related social media contents and advertising campaigns (Table 1).

**Table 1:** Social media engagement metrics by social networking platforms (2020)

Construct	Metric	Social Network
Engagement	*The no. of people a post reached who then liked, commented, shared or clicked on the post*	Facebook
Engagement Rate	$$\frac{Total no. of times a user interacted with a tweet}{{No. of Impressions}}$$	Twitter
Engagement Rate	$$\frac{No. of interactions + no. of clicks and followers}{{No. of Impressions}}$$	LinkedIn
Engagement Rate	$$\frac{No. of clicks on interactive elements}{{No. of times an ad is shown }}$$	YouTube

With the spread of social media analytics, platforms and digital service providers developed dashboards and analytical indicators to assess, measure and monitor the engagement generated by social media marketing activities (Table 2). At the same time, many bloggers, marketers, and freelancers have weighed in on the topic, enriching the debate with new contributions.

**Table 2:** Social media engagement metrics by social media management and analytics platforms (2020)

Construct	Metric	Platform
Engagement Rate	$\frac{Post Interactions}{{Number of Fans }} x 100$	Socialbakers
Engagement	$\frac{Post Interactions}{{Number of Fans}}$	Fanpage Karma
Engagement Rate	$\frac{Post Interactions}{{Total Reach }} x 100$	Talkwalker
Engagement Rate	$\frac{Post Interactions}{{Number of Impressions }}$	Hootsuite
Engagement Rate	$\frac{Post Interactions}{{Number of Impressions }}$	Dashthis
Engagement Rate	$\frac{Post Interactions}{{Number of Fans }} x 100$	Keyhole

As a result, while scholars still have to agree upon a shared definition of social media engagement, marketers have recognised it as one of the most important online outcome companies need to deliver with social media and a key metric to assess social media strategy success*.* Despite the growing interest in business practice and its solid traditional theoretical roots, most of the existing literature on social media engagement offers only conceptual guidelines (Barger et al., 2016; Peltier et al., 2020). The measurement of engagement in social media and its financial impact remains an enigma wrapped in a riddle for many executives (McKinsey, 2012) and requires further investigations. Mainly, how new and emerging platforms, new forms of customer-brand interactions, different dimensions, and cultural differences impact social media engagement measurement remains an understudied phenomenon (Peltier et al., 2020).

## Methodology

The literature review is one of the most appropriate research methods, which aims to map the relevant literature identifying the potential research gaps that need further research to contribute towards a systematic advancement of new knowledge in the field (Tranfield et al., 2003). This research is built upon the rigorous, transparent, and reproducible protocol of the systematic literature review as a scientific and transparent process that reduces the selection bias through an exhaustive literature search (Pencarelli & Mele, 2019; Pickering & Byrne, 2014; Tranfield et al., 2003). Building on recent studies (Inamdar et al., 2020; Linnenluecke et al., 2020; Phulwani et al., 2020), in addition to the systematic literature review, a bibliometric analysis (Li et al., 2017) was also performed to provide greater comprehensions into the field's current state and highlight the future research directions.

### Database, keywords, inclusion, and exclusion criteria

To conduct a literature review, quality journals are considered the basis for selecting quality publications (Wallace & Wray, 2016). Therefore, the database Scopus, run by Elsevier Publishing, was considered to search for relevant literature, being the most significant abstract and citation source database used in recent reviews.

When conducting a literature review, a fundamental issue is determining the keywords that allow identifying the papers (Aveyard, 2007). To address it, the most frequently used keywords in peer-reviewed literature have been under investigation. As such, the following research chain was used: “Social media” “Engagement” AND “metric*”, searching under title, abstract, and keywords.

**Fig. 1:**
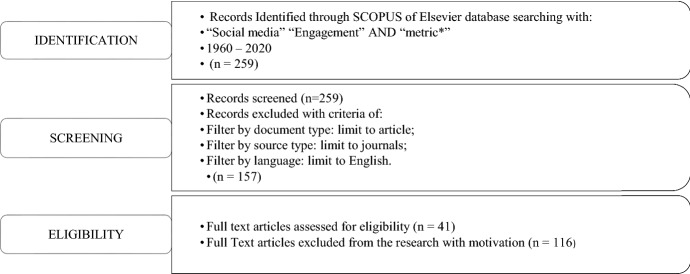
The systematic literature review protocol

The systematic literature review protocol (Fig. 1) has been conducted on the 26^th^ of March 2020. The study considers an open starting time to trace back to the origin of social media engagement metrics research up to late March 2020. The initial search attempts identified 259 documents.

After the articles’ identification, criteria for inclusion and exclusion were adopted. First, the 259 articles were screened, considering English-language articles published in peer-reviewed academic journals to safeguard the quality and effectiveness of the review. Due to variability in the peer-review process and their limited availability, book reviews, editorials, and papers from conference proceedings were excluded from this research. After the screening, a sample of 157 papers was obtained.

Afterwards, the full text of these papers was reviewed to assess eligible articles. As a result, 116 articles were excluded because their subject matter was not closely related to the topic of social media engagement metrics. In detail, papers were excluded when: 1) they mainly focused on social media engagement but superficially touched the metrics or 2) they mainly focused on metrics but superficially touched on social media engagement. In the end, 41 eligible articles were identified.

### Analysis tools

The relevant data of the 41 documents in the final sample were saved and organised in a Microsoft Excel spreadsheet to include all the essential paper information such as paper title, authors’ names, and affiliations, abstract, keywords and references. Then, adopting the bibliometrics analysis method (Aria & Cuccurullo, 2017), the R-Tool ‘Biblioshiny for Bibliometrix’ was used to perform a comprehensive bibliometric analysis. Bibliometrix is a recent R-package that facilitates a more complete bibliometric analysis, employing specific tools for both bibliometric and scientometric quantitative research (Aria & Cuccurullo, 2017; Dervis, 2019; Jalal, 2019).

## An overview of social media engagement metrics research.

The bibliometric analysis provided information on the 41 articles, allowing to highlight the significance of the topic.

### Publication trend

**Fig. 2:**
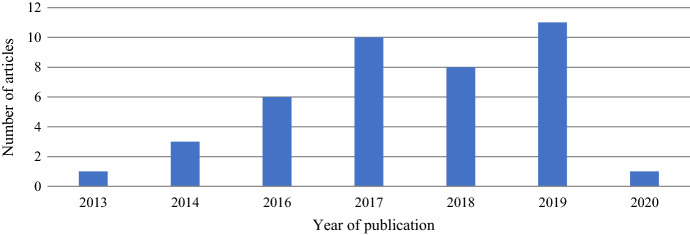
Timeline of the studies (January 2013- March 2020)

The number of annual publications shows a rollercoaster trend (Fig. 2). Although the first relevant paper was published in 2013, only since 2016 publications begun to increase significantly with a slight decrease in 2018. This renders social media engagement metrics a relatively young research field.

It is worth pointing out that the articles extraction was done in March 2020: this explains the low number of articles published in 2020.

### Most relevant sources

**Fig. 3:**
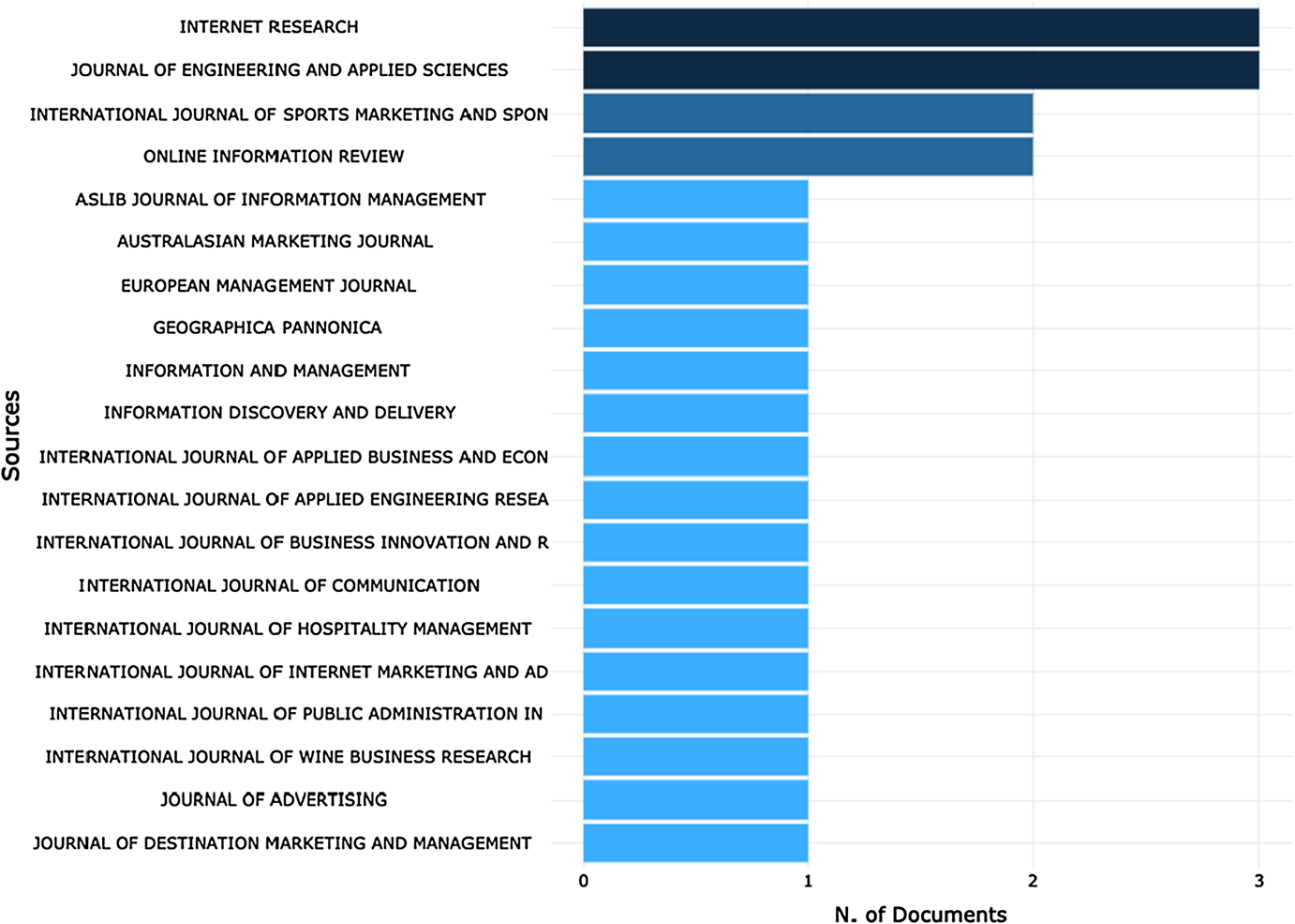
Most relevant sources

When looking at the Journal sources overview, the analysis revealed 34 journals covering different fields, including marketing, management, economics, tourism and hospitality, engineering, communication, and technology. As shown in Fig. 3, only four journals have more than two publications: *Internet Research*, *Journal of Engineering and Applied Sciences*, *International Journal of Sports Marketing and Sponsorship.* and *Online Information Review*.

### Seminal papers

Interesting findings emerged considering the most global cited documents that allow identifying the seminal articles in according to the timeliness, utility and quality, expressed by the scientific community (Okubo, 1997). The number of citations an article receives, and the studies cited in an article are two of the most popular bibliometric indicators used to determine the popularity of a publication.

**Fig. 4:**
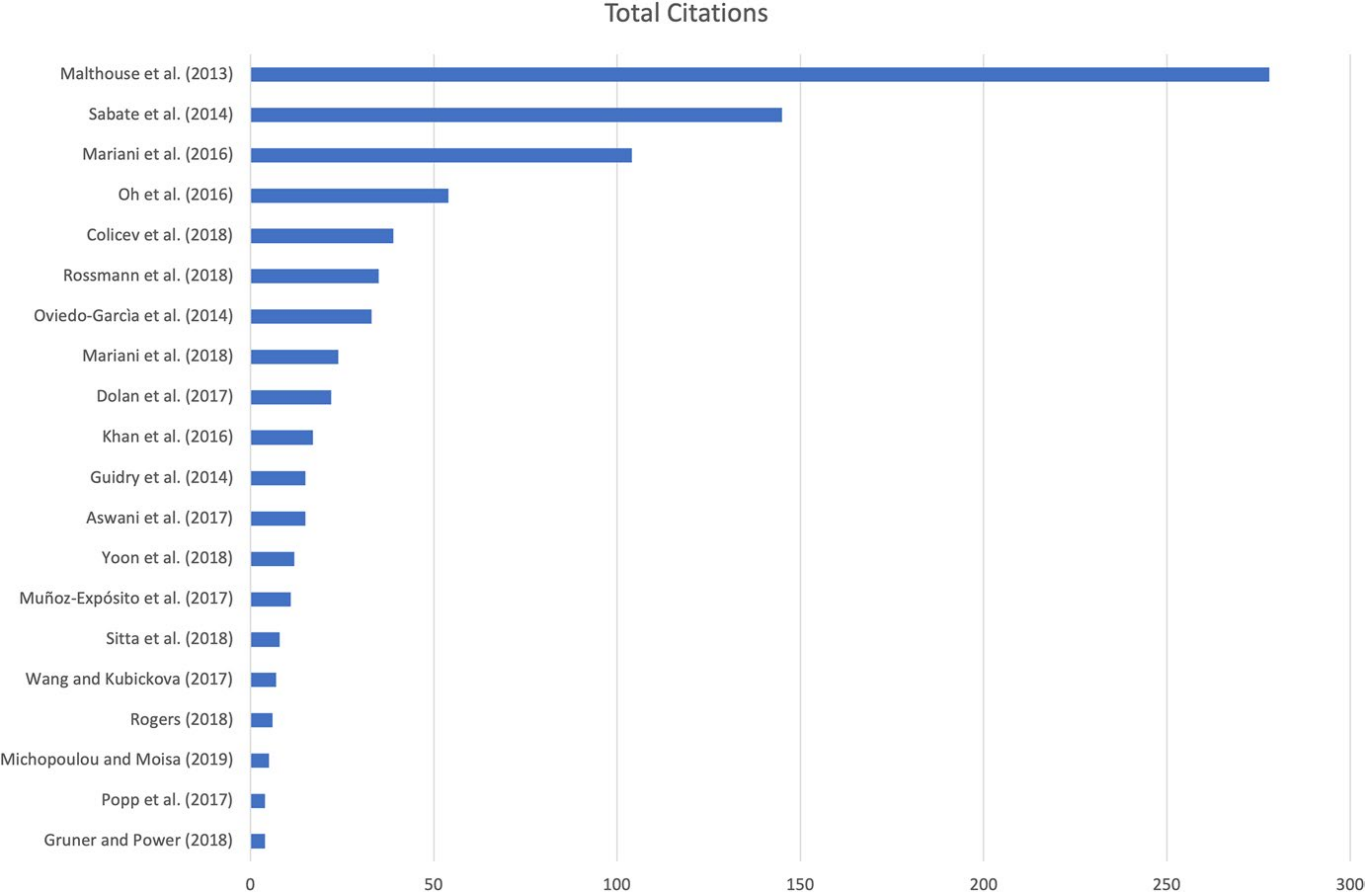
Most cited articles

Figure 4 shows the number of author citations for each article, identifying as seminal works: Malthouse’s (2013) paper ‘*Managing Customer Relationships in the Social Media Era: Introducing the Social CRM House’* with 278 global citations; Sabate’s (2014) paper ‘Factors influencing popularity of branded content in Facebook fan pages’ with 145 global citations; Mariani’s (2016) paper ‘*Facebook as a destination marketing tool: Evidence from Italian regional Destination Management Organizations*’ with 104 global citations; Oh’s (2017) paper ‘*Beyond likes and tweets: Consumer engagement behavior and movie box office in social media*’ with 54 global citations; Colicev’s (2018)’ *Improving consumer mindset metrics and shareholder value through social media: The different roles of owned and earned media*’ with 39 global citations; Rossmann’s (2016) ‘*Drivers of user engagement in eWoM communication*’ with 35 global citations; Oviedo-Garcia’s (2014) ‘*Metric proposal for customer engagement in Facebook’* with 33 global citations*.*

The analysis of the papers reviewed revealed that the theme of social media engagement metrics turns out to be a hot topic and a newly emerging stream of research.

## Social media engagement: areas of investigation

**Fig. 5:**
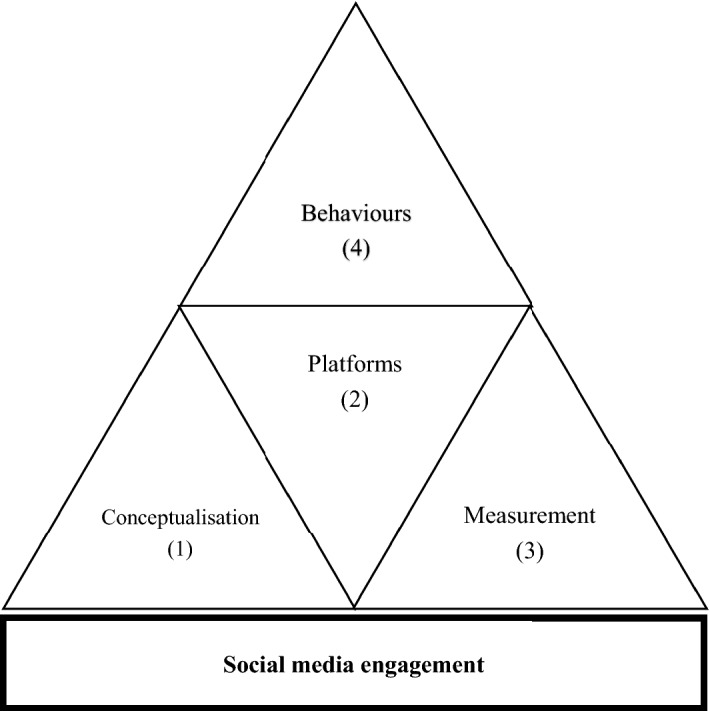
Areas of investigation

In recent years social media engagement has gained relevance in academic research, and many scholars have questioned its measurement, intensifying the academic debate with ever new contributions. Following previous studies, a comprehensive analysis allows framing the following categories of broad research subjects, used to conduct the subsequent systematic literature review (Fig. 5): (1) conceptualisation, (2) platforms, (3) measurement and (4) behaviours. All 41 articles were analysed according to the proposed scheme.

### Investigating social media engagement

What emerges from the analysis of the 41 papers is that scholars used different approaches and methodologies to conceptualise and measure engagement in the digital context of social media.

**Fig. 6:**
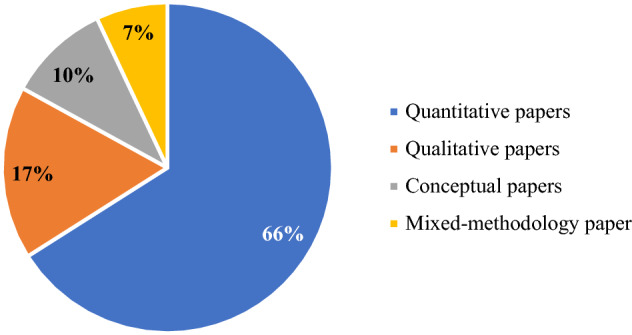
Classification of the 41 articles based on the methodology applied

As shown in Fig. 6, most studies (66%) employ quantitative methodologies. For instance, Yoon et al. (2018) explored the relationship between digital engagement metrics and financial performance in terms of company revenue, confirming that customer engagement on a company’s Facebook fan page can influence revenue. Colicev et al. (2018) developed three social media metrics, including engagement, to study the effects of earned social media and owned social media on brand awareness, purchase intention, and customer satisfaction. In comparison, Wang and Kubickova (2017) examined factors affecting the engagement metrics of Facebook fan pages in the Northeast America hotel industry, factors such as time-of-day, day-of-week, age, gender and distance between the hotel and users’ origin of residence. They also analysed the impact of Facebook engagement on electronic word-of-mouth (eWOM), to better understand the importance of the engagement metrics within the hospitality context.

From a qualitative point of view (17% of the papers), Hallock et al. (2019) used a case study approach to understand the firm perspective on social media engagement metrics, shedding light on how companies view engagement with social media as measurable metrics of customer interactions with the platform. Conversely, Michopoulou and Moisa (2019) used the same approach to investigate the use of social media marketing metrics and practices in the U.K. hotel industry.

Only a small part of the studies analysed (10% of the papers) explores social media engagement from a purely conceptual perspective. In this sense, Oviedo-Garcìa et al. (2014) and Muñoz-Expósito et al. (2017) directly identified social media engagement metrics for Facebook and Twitter, providing fascinating insights for scholars and practitioners.

Finally, among the papers analysed, only three studies (7% of the papers) use mixed methodologies to explore the phenomenon from qualitative and quantitative perspectives.

### Defining social media engagement

Researchers identified 30 unique definitions of engagement applied to the social media context. Multiple definitions used several terms when defining engagement on social media. They were not singular and straightforward but were interspersed with various key terms and overlapping concepts, as presented in Table 3.

**Table 3:** Frequency of the terms used to define engagement in social media

Terms	Frequency	%
Social media engagement	19	46%
User engagement	9	22%
Customer engagement in social media	6	15%
Online engagement	3	7%
Virtual brand community engagement	1	2.5%
Audience engagement	1	2.5%
Viewer engagement	1	2.5%
Digital engagement	1	2.5%
Total	41	100%

The presence of synonymous terms directly addresses the lack of a standard definition and the challenges that this presents to researchers and practitioners in the field (Table 4).

**Table 4:** Social media engagement main definitions

Authors	Terms	Definitions
Khan et al. (2019)	Social media engagement	*Non-monetary return from a social media investment*
Segijn et al. (2019)	Viewer engagement	*A motivation of the individual, resulting from his or her experiences with an object, which manifest in object-oriented behavior*
Hallock et al. (2019)	Social media engagement	*What occurs as a user builds relationships with other users and brands. It is more than merely liking, commenting or posting within a social network. Instead, it reveals a longer-term relationship among users or between users and a brand*
Osokin (2019)	User engagament	*Process lying within the psychological boundaries aimed at facilitating customer participation and emotional connection with a company*
Medjani et al. (2019)	Social media engagement	*Non-financial metrics Key objective of social media marketing*
Sitta et al. (2018)	Social media engagement	*A broad concept, that captures the connection consumers have with advertising, media and brands, developing creative with layers of involvement, something to participate in, something worth distributing, something to talk about*
Le et al., (2018)	Online engagement	*The consumers’ online behaviours measured by the so-called engagement metrics of online actions including number of users, click-through rates, page views, content liking and commenting depending on the platforms*
Muñoz-Expósito et al. (2017)	Customer engagement on Twitter	*The manifestation of commitment, through the intensity of interactions and their implications, toward the offers and activities of a brand, product, or firm, regardless of whether it is initiated by the individual or by the firm*
Khan et al. (2016)	Online engagement	*Attitude about brand in terms of liking, commenting and sharing*

As a relevant result, most authors focus on its behavioural manifestation (22% of the studies) resulting from motivational drivers when defining social media engagement. It is considered as the active behavioural efforts that both existing and potential customers exert toward online brand-related content (Yoon et al., 2018). It involves various activities that range from consuming content, participating in discussions, and interacting with other customers to digital buying (Oh et al., 2017; Yoon et al., 2018). Similarly, in addition to the behavioural manifestations, other scholars (12%) focus on the emotional connection expressed through the intensity of interactions and their implications, toward the offers and activities of a brand, product, or firm, regardless of whether it is initiated by the individual or by the firm (Muñoz-Expósito et al., 2017).

Shifting the observation lens from the customers to the firms, another group of scholars (10% of the studies) define social media engagement as the non-monetary return that derives from the online marketing strategies of brands (Khan, 2017; Medjani et al., 2019; Michopoulou & Moisa, 2019). In this case, engagement is viewed exclusively as a non-financial metric and as a measure of the performance of social media marketing activities.

Lastly, a small percentage of studies (10% of the studies) considers engagement as the number of people who acknowledge agreement or preference for content, who participate in creating, sharing and using content (Colicev et al., 2018; Li et al., 2019; Rahman et al., 2017).

### Social Media Platforms

In a total of 41 articles reviewed, 85% of studies mention the platforms analysed, as shown in Table 5. Facebook is the most popular platform analysed, followed by Twitter, YouTube, LinkedIn, and Instagram. These results were rather expected, given the fact that Facebook, with 2.6 billion monthly active users (Facebook, May 2020), is the most popular social media platform worldwide.

**Table 5:** Platforms mentioned in the 41 articles and related frequencies

Platform	Frequency	%
Facebook	28	52%
Twitter	12	22%
YouTube	6	11%
Instagram	1	2%
LinkedIn	1	2%
Multiple Platforms	6	11%
Total	54	100%

An interesting finding is that there are several articles (15% of the studies) which do not refer to a specific platform or that consider all the platforms together, when measuring social media engagement (e.g., Hallock et al., 2019; Medjani et al., 2019). This is interesting, given that each social network has different features that make the engagement measurement unique and not replicable.

### Measuring social media engagement

The systematic literature review confirms that there is no theoretical certainty or solid consensus among scholars about measuring engagement on social media.

As can be seen from Table 6, studies on social media engagement metrics can be grouped and classified into four macro-categories. The first group of studies, namely ‘quantitative metrics’, which is also the most numerous (66% of the studies), attempts to propose a simplistic assessment of the impact of social media engagement, based on the number of comments, likes, shares, followers etc. (Khan et al., 2019; Medjani et al., 2019; Yoon et al., 2018).

**Table 6:** Social media engagement metrics used in the 41 articles

Author	Classification	Measurement	Platform
Buffard and Papasava (2020)	Normalized Index	Unique click-through-rate	Facebook
Khan et al. (2019)	Quantitative Metrics	No. of Like, No. of Click	Facebook
Zanini et al. (2019)	Quantitative Metrics	No. of Tweet, No. of Retweet, No. of Likes, Average Tweet, Average Retweet, Average Like, Total Interactions	Twitter
Segijn et al. (2019)	Quantitative Metrics	No. of Tweets	Twitter
Hallock et al. (2019)	Quantitative Metrics	No. of users on a social platform and the regularity of their interactions on the platform	Not Specified
Osokin (2019)	Quantitative Metrics	Average no. of likes per 50 posts; Average no. of comments per 50 posts; Average no. of shares per 50 posts; No. of followers	Facebook
Kalinić and Vujičić (2019)	Quantitative Metrics	Total interactions, No. of comments, No. of shares; Average interactions, Average comments, Average shares	Facebook
Michopoulou and Moisa (2019)	Qualitative Metrics	N\A	Not Specified
Medjani et al. (2019)	Quantitative Metrics	No. of like, No. of comments, No. of shares	Not Specified
Li et al. (2019)	Set of Indexes	Conversation rate: average no. of comments per post Amplification rate: average no. of retweets and shares per post Applause rate: total no. of likes per post	Not Specified
Abuljadail and Ha, (2019)	Qualitative Metrics	Cognition, Affection, Activation	Facebook
Vrettos and Gouscos, (2019)	Set of Indexes	Popularity, Commitment, Virality, Online User Engagement	Facebook, Twitter, Youtube
Aggrawal and Arora (2019)	Quantitative Metrics	No. of like count, No. of comments, No. of shares, No. of Dislike	Youtube
McCoy et al. (2018)	Quantitative Metrics	No. of primary and secondary followers	Twitter
Mariani et al. (2018)	Set of Indexes	Generic Engagement, Brand Engagement, User Engagement, Generic Engagement for users' activity, Brand Engagement for users' activity, User Engagement for users' activity	Facebook
Sitta et al. (2018)	Quantitative Metrics	No. of follower, Monthly Growth, People Talking about this	Facebook
Yoon et al. (2018)	Quantitative Metrics	No. of Comments	Faceboo k
Rogers (2018)	Qualitative Metrics	Dominant voice, Concern, Commitment, Positioning, Alignment	Not Specified
Colicev et al. (2018)	Quantitative Metrics	People Talking about This No.of retweets, No. of video views	Facebook Twitter Youtube
Gruner and Power (2018)	Quantitative Metrics	No.of like, No.of comments People Talking about This No. of Twitter followers No. of LinkedIn followers No. of YouTube followers	Facebook Twitter LinkedIn YouTube
Le (2018)	Quantitative Metrics	No. of like, No. of comments, No. of shares	Facebook
Aswani et al. (2017)	Set of Indexes	Number of created discussions, Author increase, Attention paid, Burstiness level	Twitter
Rahman et al. (2017)	Quantitative Metrics	No. of like, No. of comments, No. of shares	Facebook
Oh et al. (2017)	Quantitative Metrics	People Talking about This No. of Facebook followers No. of Tweet No. of YouTube Views No. of YouTube Comments	Facebook Twitter YouTube
Dolan et al. (2017)	Normalized Index	The percentage of people who saw a post (post reach) and liked, shared, clicked or commented on it	Facebook
Rahman, et al. (2017)	Quantitative Metrics	No. of like, No. of comments, No. of shares	Facebook
Rahman, et al. (2017)	Quantitative Metrics	No. of like, No. of comments, No. of shares	Facebook
Popp et al. (2017)	Quantitative Metrics	No. of Facebook Followers, No. of Twitter Followers	Facebook Twitter
Muñoz-Expósito, et al. (2017)	Normalized Index	Engagement on Twitter [(Interactions\No. of tweets) \ (Average impressions)]\ (Average reach) * 100	Twitter
Wang and Kubickova (2017)	Normalized Index	Daily Engaged users; Lifetime Engaged Users; People Talking About This	Facebook
Vlachvei and Kyparissis (2017)	Normalized Index	$\frac{{{\text{Like}} + {\text{Comments}} + {\text{Shares}}}}{{\text{Number of Followers }}}$	Facebook
Rossmann et al. (2016)	Normalized Index	$\frac{{{\text{Like}} + {\text{Comments}}}}{{\text{Reach }}}{ }$	Facebook
Mariani et al. (2016)	Set of Indexes	Generic Engagement, Brand Engagement, User Engagement, Generic Engagement for users' activity, Brand Engagement for users' activity, User Engagement for users' activity	Facebook
Rahman et al. (2016)	Quantitative Metrics	No. of like, No. of comments, No. of shares	Facebook
Khan et al. (2016)	Quantitative Metrics	No. of like, No. of comments, No. of shares	Facebook
Rahman et al. (2016)	Quantitative Metrics	No. of like, No. of comments, No. of shares	Facebook
Rahman et al. (2016)	Quantitative Metrics	No. of like, No. of comments, No. of shares	Facebook
Sabate et al. (2014)	Quantitative Metrics	No. of like, No. of comments	Facebook
Oviedo-García et al. (2014)	Normalized Index	Engagement on Facebook = [(Like + Comment + share\ number of post) \Average Impressions] \average reach	Facebook
Guidry et al. (2014)	Quantitative Metrics	No. of like, No. of retweet, No. of click on hyperlink, No. tweet resulted in conversation	Twitter
Malthouse et al. (2013)	Quantitative Metrics	Lower customer engagement No. of like, No. of comments, No. of views, No. of Click Higher customer engagement No. of Review, No. of Shares	Not Specified

The second group of studies (17% of the studies), namely ‘normalised indexes’, provide a quantitative evaluation of the engagement a content generates in relation to the number of people to whom that content has been displayed. In this way, it is possible to obtain an average measure of the users’ engagement, dividing the total actions of interest by the total number of posts (Osokin, 2019; Zanini et al., 2019), the number of followers (Vlachvei & Kyparissi, 2017) or the number of people reached by a post (Muñoz-Expósito et al., 2017; Rossmann et al., 2016).

In a more complex and detailed way, studies from the third group (10% of the studies) identify social media engagement metrics developing ‘set of indexes’. For example, Li et al. (2019) use three social media metrics to measure engagement in the casual-dining restaurant setting: rates of conversation, amplification, and applause. In detail, conversation rate measures the number of comments or reviews in response to a post, amplification rate measures how much online content is shared, and applause rate measures the number of positive reactions on posts. Similarly, drawing from previous literature, Mariani et al. (2018) develop three social media metrics, namely generic engagement, brand engagement, and user engagement. Authors calculated these metrics by assessing different weights to different interaction actions, to emphasise the degree of users’ involvement implied by the underlying activities of respectively liking, sharing, or commenting.

Despite their great diffusion among academics and practitioners, some scholars (7% of the studies) argue that quantitative metrics are not enough to appreciate the real value of customer engagement on social media, and a qualitative approach is more suitable. For example, Abuljadail and Ha (2019) conducted an online survey of 576 Facebook users in Saudi Arabia to examine customer engagement on Facebook. Rogers (2018) critiques contemporary social media metrics considered ‘vanity metrics’ and repurpose alt metrics scores and other engagement measures for social research—namely dominant voice, concern, commitment, positioning, and alignment—to measure the ‘otherwise engaged’.

### Social media engagement brand-related activities

When measuring social media engagement, scholars dealt with different social media actions that can be classified (Table 7) according to the three dimensions of the COBRA model (Consumer Online Brand Related Activities): consumption, contribution, or creation (Schivinski et al., 2016).

**Table 7:** Dimensions of the COBRA model and related frequencies

Dimensions of the COBRA model	Frequency	%
Consumption	7	14%
Contribution	35	68%
Creation	9	18%
Total	51	100%

In a total of 41 articles reviewed, the most investigated dimension by researchers is contribution, i.e. when a customer comments, shares, likes a form of pre-existing brand content (e.g., Buffard et al., 2020; Khan et al., 2019). Its popularity among the studies may be due to its interactive nature of “liking” and “commenting”, which can be said to be the most common behaviour exhibited across social media platforms and often one of the most manageable interactions to obtain data. Additionally, studies that include creation in the measurement of social media engagement consider posting/publishing brand-related content, uploading brand-related video, pictures, audio or writing brand-related articles (e.g., Zanini et al., 2019). Among the sampled papers, the least investigated dimension of the COBRA model is consumption, considered by only seven studies (e.g., Colicev et al., 2018; Oh et al., 2017). It considers viewing brand-related audio, video, and pictures, following threads on online brand community forums or downloading branded widgets.

Dimensions have been investigated individually, for example, just considering the number of likes or comments (Khan et al., 2019; Yoon et al., 2018), or jointly using composite indicators, as in the case of Oviedo-Oviedo-García et al., 2014).

## Discussion

This research presents fresh knowledge in the academic debate by providing an overarching picture of social media engagement, framing the phenomenon conceptually and offering a lens to interpret platforms and measuring tools. Conceptual and empirical studies tried to define, conceptualise, and measure social media engagement in diverse ways from different fields of research. They increased the gap between academia and managerial practice, where the topic of social media engagement metrics seems to be much more consolidated. The paper contributes to the academic debate on social media engagement, presenting continuity and discontinuity elements between different fields of enquiry. It also offers avenues for future research that both academics and marketers should explore. It also provides insights and guidance to practitioners on modelling and managing social media engagement.

### Theoretical contribution

The article offers some theoretical contributions to this relatively young research field through the systematic literature review approach.

Firstly, the paper confirms the multidimensional and polysemic nature of engagement, even in the specific context of social media platforms, in continuity with the academic customer engagement research (Brodie et al., 2013; Hollebeek et al., 2016; So et al., 2016; Vivek et al., 2012). The concept of social media engagement can be traced back to three dimensions of analysis (Van Doorn, 2010)—affective, cognitive, and behavioural—and some empirical studies measure it as such (Dessart, 2017; Vivek et al., 2014). However, the behavioural dimension is still the most used proxy to measure users’ level of engagement. Similarly, marketers and social media platforms have focused on behavioural interactions associated with likes, comments and sharing when reporting engagement metric (Peltier et al., 2020). What is worth pointing out is that emotional and cognitive dimensions are also essential components of social media engagement and should be adequately addressed by future research.

Secondly, strictly related to the first point, the paper suggests the COBRA model (Schivinski, 2016) as a conceptual tool to classify and interpret social media engagement from the behavioural perspective. Social media engagement can be manifested symbolically through actions (Barger et al., 2016; Oh et al., 2017; Van Doorn et al., 2010) that can be traced back to the three dimensions of consumption, contribution and creation (Schivinski et al., 2016). However, it is worth pointing out that not all these actions determine the same level of engagement. When measuring social media engagement, researchers should pay attention not only to ‘contribution’ but also to ‘consumption’ and ‘creation’, which are important indicators of the attention a post receives (Oviedo-Garcìa, 2014; Schivinski et al., 2016), giving them a different weight. It becomes even more important if considering that the same social networks provide different weights to users' actions. For example, in several countries, Instagram has tested removing the like feature on content posted by others, although users can still see the number of likes on their posts. YouTube has also decided to stop showing precise subscriber counts and Facebook is experimenting with hiding like counts, similar to Instagram.

Thirdly, the paper presents some of the key metrics used to evaluate social media engagement identifying quantitative metrics, normalised indexes, set of indexes and qualitative metrics. Although all indicators are based on the interaction between the user and the brand, as the literature suggests (Barger et al., 2016; Oviedo-Garcìa, 2014; Vivek et al., 2014), the paper argues that different metrics measure diverse aspects of social media engagement and should be used carefully by researchers. Despite the conceptual and qualitative research on the topic, even the most recent metrics offer measurements that do not allow engagement to be widely represented in its multidimensional and polysemic nature (Oviedo-García et al., 2014; Peltier et al., 2020). To get a deeper understanding of the construct, researchers should also consider some of the most recent advances in business practice. As an example, more and more practitioners have the chance to measure engagement by tracking the time spent on content and web pages to blend the different types of material, such as pictures, text, or even videos. Also, cursor movements, which are known to correlate with visual attention, and eye-tracking, can provide insights into the within-content engagement.

### Managerial implications

Even if the topic of social media engagement seems to be more consolidated in business practice, this study also provides valuable implications for practitioners. Particularly, the findings shed light on the nature of social media engagement construct and on how metrics can be an extremely useful tool to evaluate, monitor, and interpret the effectiveness of social media strategies and campaigns.

This research offers a strategic-operational guide to the measurement of social media engagement, helping marketers understand what engagement is and choose the most effective and suitable KPIs to assess the performance and success of their marketing efforts. In this sense, marketers should accompany traditional metrics, such as likes, comments and shares, with new metrics capable of better capturing user behaviours.

Marketers also need to realise that engagement is a complex construct that goes beyond the simple behavioural dimension, encompassing cognitive and emotional traits. As a result, in some cases, the so-called “vanity metrics” could fail in fully representing all the aspects of social media engagement. In these cases, it should be accompanied by qualitative insights to analyse what users like to share or talk about and not merely look at likes, comments, and shares counts.

## Limitations and future research

This research is not without limitations. First, the systematic literature review only includes English articles published in Journals. As social media engagement and engagement metrics are emerging research topics, conference proceedings and book chapters could also be included to deepen the understanding of the subject. Second, this research was conducted on the database Scopus of Elsevier for the keywords “social media engagement metrics”. Researchers could use a combination of different databases and keywords to search for new contributions and insights. Third, although the paper is based on a systematic literature review, this methodology reveals the subjectivity in the social sciences.

As this is a relatively young field of research, a further academic investigation is needed to overcome the limitations of the study and outline new scenarios and directions for future research. In addition, considering the growing importance of social media, there is value in broadening the analysis through additional studies. Future marketing research could use mixed approaches to integrate the three dimensions of social media engagement, linking qualitative and quantitative data. Advanced sentiment web mining techniques could be applied to allow researchers to analyse what users like to share or talk about and not merely look at likes, comments, and shares as the only metrics (Peltier et al., 2020).

Although Facebook and Twitter are the most used social network by brands, and the most significant part of the literature focuses on these two platforms, researchers should not forget that there are new and emerging social media in different countries (e.g., TikTok, Clubhouse). They already represent a hot topic for practitioners and are calling scholars to define new metrics to measure engagement. Additionally, as the use of social media increased during the COVID-19 pandemic, future research should take this into account to better understand social media engagement across different social media platforms.

## References

[CR1] Abuljadail M, Ha L (2019). Engagement and brand loyalty through social capital in social media. International Journal of Internet Marketing and Advertising.

[CR2] Advertising Research Foundation. (2006). *Engagement: Definitions and Anatomy*. ARF White Paper. https://thearf.org/. Retrieved 5 May 2021

[CR3] Aggrawal N, Arora A (2019). Behaviour of viewers: YouTube videos viewership analysis. International Journal of Business Innovation and Research.

[CR4] Aria M, Cuccurullo C (2017). Bibliometrix: An R-tool for comprehensive science mapping analysis. Journal of Informetrics.

[CR5] Aswani R, Ghrera SP, Kar AK, Chandra S (2017). Identifying buzz in social media: A hybrid approach using artificial bee colony and k-nearest neighbors for outlier detection. Social Network Analysis and Mining.

[CR6] Aveyard H (2007). Doing a Literature Review in Health and Social Care: A practical guide.

[CR7] Barger V, Peltier JW, Schultz DE (2016). Social media and consumer engagement: A review and research agenda. Journal of Research in Interactive Marketing.

[CR8] Bowden J (2009). The process of customer engagement: A conceptual framework. Journal of Marketing Theory and Practice.

[CR9] Brodie RJ, Hollebeek LD, Jurić B, Ilić A (2011). Customer engagement: Conceptual domain, fundamental propositions, and implications for research. Journal of Service Research.

[CR10] Brodie RJ, Ilic A, Juric B, Hollebeek L (2013). Consumer engagement in a virtual brand community: An exploratory analysis. Journal of Business Research.

[CR11] Buffard J, Papasava A (2020). A quantitative study on the impact of emotion on social media engagement and conversion. Journal of Digital and Social Media Marketing.

[CR12] Colicev A, Malshe A, Pauwels K, O’Connor P (2018). Improving consumer mindset metrics and shareholder value through social media: The different roles of owned and earned media. Journal of Marketing.

[CR15] De Vries NJ, Carlson J (2014). Examining the drivers and brand performance implications of customer engagement with brands in the social media environment. Journal of Brand Management.

[CR16] Dervis H (2019). Bibliometric analysis using bibliometrix an R package. Journal of Scientometric Research.

[CR17] Dessart L (2017). Social media engagement: A model of antecedents and relational outcomes. Journal of Marketing Management.

[CR19] Dolan R, Conduit J, Fahy J, Goodman S (2017). Social media: Communication strategies, engagement and future research directions. International Journal of Wine Business Research.

[CR13] Forrester Consulting. (2008). *How engaged are your customers?*. Forrester Consuting. http://docplayer.net/9663683-How-engaged-are-your-customers.html. Retrieved 5 May 2021

[CR14] Gallup Consulting. (2009). *Customer engagement: What’s your engagement ratio?*. Gallup Consulting. https://strengthszone.com/wp-content/uploads/2016/01/Customer-Engagement-Ratio-Brochure.pdf. Retrieved 5 May 2021

[CR20] Gruner RL, Power D (2018). To integrate or not to integrate? Understanding B2B social media communications. Online Information Review.

[CR21] Guidry JPD, Waters RD, Saxton GD (2014). Moving social marketing beyond personal change to social change: Strategically using Twitter to mobilize supporters into vocal advocates. Journal of Social Marketing.

[CR22] Hallock W, Roggeveen AL, Crittenden V (2019). Firm-level perspectives on social media engagement: An exploratory study. Qualitative Market Research.

[CR23] Harrigan P, Evers U, Miles M, Daly T (2017). Customer engagement with tourism social media brands. Tourism Management.

[CR24] Haumann T, Güntürkün P, Schons LM (2015). Engaging customers in coproduction processes: How value-enhancing and intensity-reducing communication strategies mitigate the negative effects of coproduction intensity. Journal of Marketing.

[CR88] Hollebeek LD (2018). Individual-level cultural consumer engagement styles: Conceptualization, propositions and implications. International Marketing Review.

[CR25] Hollebeek LD (2019). Developing business customer engagement through social media engagement-platforms: An integrative S-D logic/RBV-informed model. Industrial Marketing Management.

[CR26] Hollebeek LD, Conduit J, Brodie RJ (2016). Strategic drivers, anticipated and unanticipated outcomes of customer engagement. Journal of Marketing Management.

[CR27] Hollebeek LD, Glynn MS, Brodie RJ (2014). Consumer brand engagement in social media: Conceptualization, scale development and validation. Journal of Interactive Marketing.

[CR28] Hollebeek LD, Srivastava RK, Chen T (2019). S-D logic–informed customer engagement: Integrative framework, revised fundamental propositions, and application to CRM. Journal of the Academy of Marketing Science.

[CR29] Hubspot. (2014). *CRM expert Paul Greenberg defines customer engagement*. Hubspot. https://blog.hubspot.com/sales/paul-greenberg-defines-customer-engagement. Retrieved 5 May 2021

[CR30] Inamdar Z, Raut R, Narwane VS, Gardas B, Narkhede B, Sagnak M (2020). A systematic literature review with bibliometric analysis of big data analytics adoption from period 2014 to 2018. Journal of Enterprise Information Management.

[CR31] Jaakkola E, Alexander M (2014). The role of customer engagement behavior in value co-creation: A service system perspective. Journal of Service Research.

[CR32] Jalal SK (2019). Co-authorship and co-occurrences analysis using bibliometrix r-package: A case study of india and bangladesh. Annals of Library and Information Studies.

[CR33] Kalinić Č, Vujičić M (2019). A subnational assessment of hotel social media metrics - The case of Serbia. Geographica Pannonica.

[CR34] Khan G, Mohaisen M, Trier M (2019). The network ROI: Concept, metrics, and measurement of social media returns (a Facebook experiment). Internet Research.

[CR35] Khan I, Dongping H, Wahab A (2016). Does culture matter in effectiveness of social media marketing strategy? An investigation of brand fan pages. Aslib Journal of Information Management.

[CR36] Khan ML (2017). Social media engagement: What motivates user participation and consumption on YouTube?. Computers in Human Behavior.

[CR37] Kumar V, Aksoy L, Donkers B, Venkatesan R, Wiesel T, Tillmanns S (2010). Undervalued or overvalued customers: Capturing total customer engagement value. Journal of Service Research.

[CR38] Kumar V, Rajan B, Gupta S, Dalla Pozza I (2019). Customer engagement in service. Journal of the Academy of Marketing Science.

[CR39] Le TD (2018). Influence of WOM and content type on online engagement in consumption communities : The information flow from discussion forums to Facebook. Online Information Review.

[CR40] Li J, Kim WG, Choi HM (2019). Effectiveness of social media marketing on enhancing performance: Evidence from a casual-dining restaurant setting. Tourism Economics.

[CR41] Li X, Wu P, Shen GQ, Wang X, Teng Y (2017). Mapping the knowledge domains of building information modeling (BIM): A bibliometric approach. Automation in Construction.

[CR42] Linnenluecke MK, Marrone M, Singh AK (2020). Conducting systematic literature reviews and bibliometric analyses. Australian Journal of Management.

[CR43] Liu X, Shin H, Burns AC (2019). Examining the impact of luxury brand’s social media marketing on customer engagement: Using big data analytics and natural language processing. Journal of Business Research.

[CR44] Loureiro SMC, Gorgus T, Kaufmann HR (2017). Antecedents and outcomes of online brand engagement: The role of brand love on enhancing electronic-word-of-mouth. Online Information Review.

[CR45] Malthouse EC, Haenlein M, Skiera B, Wege E, Zhang M (2013). Managing customer relationships in the social media era: Introducing the social CRM house. Journal of Interactive Marketing.

[CR46] Mariani MM, Di Felice M, Mura M (2016). Facebook as a destination marketing tool: Evidence from Italian regional destination management organizations. Tourism Management.

[CR47] Mariani MM, Mura M, Di Felice M (2018). The determinants of Facebook social engagement for national tourism organizations’ Facebook pages: A quantitative approach. Journal of Destination Marketing and Management.

[CR52] Marketing Science Institute. (2020). *Research priorities 2020–2022*. Marketing Science Institute. https://www.msi.org/wp-content/uploads/2020/06/MSI_RP20-22.pdf. Retrieved 5 May 2021

[CR48] McCoy CG, Nelson ML, Weigle MC (2018). Mining the Web to approximate university rankings. Information Discovery and Delivery.

[CR18] McKinsey. (2012). *Demystifyng social media*. McKinsey. https://www.mckinsey.com/business-functions/marketing-and-sales/our-insights/demystifying-social-media. Retrieved 5 May 2021

[CR49] Medjani F, Rutter R, Nadeau J (2019). Social media management, objectification and measurement in an emerging market. Business and Emerging Markets.

[CR50] Michopoulou E, Moisa DG (2019). Hotel social media metrics: The ROI dilemma. International Journal of Hospitality Management.

[CR53] Muñoz-Expósito M, Oviedo-García MÁ, Castellanos-Verdugo M (2017). How to measure engagement in Twitter: Advancing a metric. Internet Research.

[CR54] Muntinga DG, Moorman M, Smit EG (2011). Introducing COBRAs: Exploring motivations for brand-related social media use. International Journal of Advertising.

[CR55] Oh C, Roumani Y, Nwankpa JK, Hu HF (2017). Beyond likes and tweets: Consumer engagement behavior and movie box office in social media. Information and Management.

[CR56] Okubo Y (1997). Bibliometric indicators and analysis of research systems: methods and examples.

[CR57] Osokin N (2019). User engagement and gratifications of NSO supporters on Facebook: Evidence from European football. International Journal of Sports Marketing and Sponsorship.

[CR58] Oviedo-García MÁ, Muñoz-Expósito M, Castellanos-Verdugo M, Sancho-Mejías M (2014). Metric proposal for customer engagement in Facebook. Journal of Research in Interactive Marketing.

[CR60] Peltier J, Dahl AJ, VanderShee BA (2020). Antecedent consumer factors, consequential branding outcomes and measures of online consumer engagement: Current research and future directions. Journal of Research in Interactive Marketing.

[CR61] Pencarelli T, Mele G (2019). A systematic literature review on social media metrics. Mercati & Competitività.

[CR62] Phulwani PR, Kumar D, Goyal P (2020). A Systematic Literature Review and Bibliometric Analysis of Recycling Behavior. Journal of Global Marketing.

[CR86] Pickering C, Byrne J (2014). The benefits of publishing systematic quantitative literature reviews for PhD candidates and other early-career researchers. Higher Education Research & Development.

[CR63] Popp N, McEvoy C, Watanabe N (2017). Do college athletics marketers convert social media growth into ticket sales?. International Journal of Sports Marketing and Sponsorship.

[CR64] Rahman Z, Suberamanian K, Zanuddin H, Moghavvemi S, Bin MdNasir MHN (2016). SNS metrics analysis “A study on fanpage interactive contents”. International Journal of Applied Business and Economic Research.

[CR65] Rahman Z, Suberamanian K, Zanuddin H, Moghavvemi S, Nasir MHNM (2017). Fanpage viral metrics analysis “study on frequently posted contents”. Journal of Engineering and Applied Sciences.

[CR66] Rather RA, Hollebeek LD, Islam JU (2019). Tourism-based customer engagement: The construct, antecedents, and consequences. The Service Industries Journal.

[CR67] Rietveld R, Van Dolen W, Mazloom M, Worring M (2020). What you feel, is what you like influence of message appeals on customer engagement on Instagram. Journal of Interactive Marketing.

[CR68] Rogers R (2018). Digital traces in context| Otherwise engaged: Social media from vanity metrics to critical analytics. International Journal of Communication.

[CR69] Rossmann A, Ranjan KR, Sugathan P (2016). Drivers of user engagement in eWoM communication. Journal of Services Marketing.

[CR70] Sabate F, Berbegal-Mirabent J, Cañabate A, Lebherz PR (2014). Factors influencing popularity of branded content in Facebook fan pages. European Management Journal.

[CR71] Schivinski B, Christodoulides G, Dabrowski D (2016). Measuring consumers’ engagement with brand-related social-media content: Development and validation of a scale that identifies levels of social-media engagement with brands. Journal of Advertising Research.

[CR72] Segijn CM, Maslowska E, Araujo T, Viswanathan V (2019). Engaging with TV events on Twitter: The interrelations between TV consumption, engagement actors, and engagement content. Internet Research.

[CR73] Sitta D, Faulkner M, Stern P (2018). What can the brand manager expect from Facebook?. Australasian Marketing Journal.

[CR74] So KKF, King C, Sparks BA, Wang Y (2016). Enhancing customer relationships with retail service brands: The role of customer engagement. Journal of Service Management.

[CR75] Tranfield D, Denyer D, Smart P (2003). Towards a methodology for developing evidence-informed management knowledge by means of systematic review. British Journal of Management.

[CR76] Trunfio M, Della Lucia M (2019). Engaging destination stakeholders in the digital era: The best practice of italian regional DMOs. Journal of Hospitality and Tourism Research.

[CR77] Van Doorn J, Lemon KN, Mittal V, Nass S, Pick D, Pirner P, Verhoef PC (2010). Customer engagement behavior: Theoretical foundations and research directions. Journal of Service Research.

[CR87] Vivek SD, Beatty SE, Dalela V, Morgan RM (2014). A generalized multidimensional scale for measuring customer engagement. Journal of Marketing Theory and Practice.

[CR78] Vivek SD, Beatty SE, Morgan RM (2012). Customer engagement: Exploring customer relationships beyond purchase. Journal of Marketing Theory and Practice.

[CR79] Vlachvei A, Kyparissis A (2017). Museums on Facebook wall: A case staudy of Thessaloniki’s museums. Tourismos.

[CR80] Vrettos K, Gouscos D (2019). Evaluating the presence of Greek tourism-related public sector entities in online social networks. International Journal of Public Administration in the Digital Age.

[CR81] Wallace M, Wray A (2016). Critical reading and writing for postgraduates.

[CR82] Wang C, Kubickova M (2017). The impact of engaged users on eWOM of hotel Facebook page. Journal of Hospitality and Tourism Technology.

[CR83] Wu J, Fan S, Zhao JL (2018). Community engagement and online word of mouth: An empirical investigation. Information & Management.

[CR84] Yoon G, Li C, Ji Y, North M, Hong C, Liu J (2018). Attracting comments: digital engagement metrics on facebook and financial performance. Journal of Advertising.

[CR85] Zanini MT, Carbone de Moraes F, Lima V, Migueles C, Lourenco C, Reis Irigaray HA (2019). Soccer and twitter: Virtual brand community engagement practices. Marketing Intelligence and Planning.

